# Soil Fungi in Sustainable Agriculture

**DOI:** 10.3390/microorganisms12010163

**Published:** 2024-01-13

**Authors:** Nikolay Vassilev, Gilberto de Oliveira Mendes

**Affiliations:** 1 Department of Chemical Engineering, University of Granada, C/Fuentenueva s/n, 18071 Granada, Spain; 2 Institute of Biotechnology, University of Granada, C/Fuentenueva s/n, 18071 Granada, Spain; 3 Laboratório de Microbiologia e Fitopatologia, Instituto de Ciências Agrárias, Universidade Federal de Uberlândia, Monte Carmelo 38500-000, Brazil; gilbertomendes@ufu.br

## 1. General Remarks on the Importance of Soil Fungi

It is widely accepted that the continuously growing human population needs rapid solutions to respond to the increased global demand for high agricultural productivity. These efforts should, at the same time, follow the principles of sustainability and circular economy. It is well known that a gram of undisturbed soil contains thousands of individual microbial taxa including bacteria, fungi, protists, oomycetes and viruses, which determine soil fertility and enhance plant growth and health [1]. The best and most logical way to improve soil fertility and increase plant growth is to use and manage plant beneficial microorganisms [2]. Until recently, chemical fertilization was widely used in soil–plant systems, but this resulted in a decrease in soil natural fertility, plant diversity and microbial richness [3]. In addition to the overload of chemical fertilizers, an increasing number of stress factors, such as salinity, alkalinity/acidity, contamination, nutrient deficiency or drought, soil disturbance due to climate change, and various biotic factors, are affecting the overall soil–plant characteristics [4]. The use of plant-beneficial microorganisms, including combinations of pro- and postbiotics, is now a common practice applied to manage and stimulate the existing beneficial microbiome to mitigate all these problems in plant production, bearing also in mind the principles of modern, sustainable agriculture in the conditions of the increasing world population and environmental and climate changes [5]. During the last few years, a large number of plant-beneficial microorganisms have been isolated and tested in controlled and natural conditions. The results confirmed the beneficial effect of the selected microorganisms on plant growth and health, enhancing nutrient content and improving soil properties. Among soil microbes, fungal communities play important roles in agriculture and the soil environment. Many fungal microorganisms are known for their potential biotechnological applications including in agriculture as they synthesize functional bioactive compounds for plant growth promotion and serve as biocontrol agents. Within the vast fungal diversity associated with plant systems, arbuscular mycorrhizal fungi (AMF) occupy a special place due to their almost universal soil occupation [6] and role in plant nutrition and health [7], particularly in conditions of the changing climate [8] and soils with very low fertility due to contamination [9] or desertification [10].

## 2. Short Notes on the Contributions to the Special Issue “Soil Fungi in Sustainable Agriculture”

The first two contributions of the Special Issue describe the effect of AMF on different plant species grown in disturbed soils. AM fungi serve as a bridge between soil and plants, exchanging nutrients through their specific symbiotic relations with plants. Although they are commonly present in soils and are found in 80% of the plants, their density, diversity, and composition in agricultural environments may be limited due to different agricultural practices and soil manipulations. Such practices lead to agricultural disturbance, which in turn results in ineffective AM fungal communities. The introduction of formulated single or multiple microbial AM-based inoculants [11] into conventional exhausted and/or organic soils is accepted as a useful approach to the management of disturbed soils. The **first contribution** showed that applications of native fungi isolated from old-growth ecosystems can be characterized with increased beneficial functions and can benefit plants grown in environments such as restored soils and organic agriculture. Restoration of degraded sagebrush steppe was the aim of the **second contribution**. Applying AMF-bearing soil from a disturbed site, soil from an undisturbed site, and commercial AMF inoculum, all with and without biochar, the growth and AMF root colonization of three test plants were analyzed. The plants responded positively to all the treatments, but the analyzed parameters were different in different plants depending on the type of the inoculum. Biochar was shown to decrease the growth but increased AMF colonization.

Mycorrhizal fungi extend their extensive networks of hyphae into the soil, greatly expanding the surface area for nutrient uptake and plants colonized by AMF easily access phosphorus and other essential nutrients that would otherwise be unavailable. However, filamentous fungi, producing organic acids, are the most efficient P-solubilizers [12] with simultaneous “side effects” including biocontrol [13] and the possibility of applying compatible double inoculants or cell-free (postbiotic) biofertilizer [14,15]. Fungi can also promote plant tolerance to various environmental stresses, such as heavy metals, salinity, and drought. They help plants adapt to these harsh conditions by providing nutrients, improving water uptake, and regulating stress hormones [16]. In the **third contribution**, the authors describe the whole process of production of plant-available P by soil microorganisms, which is extremely complex with a wide number of abiotic and biotic factors characterizing each different soil or site. For this reason, there is no universal fungal biofertilizer able to provoke efficient P-solubilization and high plant growth enhancement everywhere. However, one of the most potent microbial P-solubilizers able to solubilize insoluble P-sources, including in highly weathered soils, is *Aspegillus niger* [17]. In the **fourth contribution**, the authors showed that seedlings inoculated with *A. niger* demonstrated significantly higher growth than uninoculated ones using different test crops regardless of the inoculant dose and inoculation method. The highest relative increase promoted by the fungus was observed for above-ground parts, increasing the production of shoot fresh mass of all the plants. Another important point of this contribution is the fact that *A. niger* was effective at the different doses of conidia (10^2^ and 10^6^ conidia plant^−1^) with a high germination rate, thus confirming previous findings concerning fungal spore germination [18].

In soil–plant management practices, phosphate-solubilizing microorganisms are now considered as a new alternative to phosphate chemical fertilizers and microorganisms such as *Aspergillus*, *Penicillium*, and *Pseudomonas* are repeatedly shown to solubilize insoluble organic and inorganic P by decreasing the pH and/or releasing phosphatases in the surrounding environment. However, other fungi have also been characterized by high P-solubilizing efficiency as shown by the **fifth contribution**. Four P-solubilizing microorganisms were isolated from the rhizosphere soil of a poplar plantation and further characterized as *Mortierella*. One of them demonstrated high organic acid and phosphatase production and could be applied as an organic and inorganic P solubilizer. Bearing in mind that some *Mortierella* spp. have well-expressed potential biocontrol functions exerting inductive effects, which increase plant resistance to a variety of pathogens [19], the potential of these fungi seems unexploited.

The current agricultural practice includes the wide use of pesticides that diminishes the crop infestations, thus limiting the crop harvest losses. However, the pesticides and their toxic degradation products can enter the food chain or remain in the soil and water environments, negatively affecting the soil fertility [20]. Therefore, the development of ecofriendly technologies involves the utilization of indigenous microflora, which can degrade/transform pesticides via co-metabolism or mineralization [21]. Most of the recent comprehensive reviews on the biodegradation of pesticides focus primarily on bacterial interaction with pesticides, while fungi are mentioned only marginally. However, **contribution 6** highlighted that fungi possess high potential to degrade recalcitrant organic pollutants in the environment due to their ability to exude lignolytic extracellular enzymes and acidic metabolites. The authors analyzed the potential of various *Aspergillus* and *Penicillium* species to degrade recalcitrant, persistent, and toxic pollutants in laboratory conditions underlying the need of field applications. Another important task is to carry out further studies with these filamentous fungi in complex microbial communities. The latter point is also discussed in **contribution 7**, but the study was focused on changes in the fungal community in the soil–tomato system. According to the reported data, the continued organic fertigation after basal manure application does not impact soil fungal communities, tomato yield, or soil fertility. The application of fresh sheep manure as a basal fertilizer in intensive tomato crops is proposed as a crucial practice in order to achieve sustainable management of vegetable growth in greenhouse agriculture.

## 3. Conclusions

Fungi are relatively understudied, and we know very little not only regarding specific fungal interactions with other soil organisms but also the factors that determine the fungal role in plant–microbial interactions. We should study more and learn how to explore fungi more effectively in the frame of sustainable agriculture following the 3-P strategy (prebiotics, probiotics, and postbiotics). The main lines of study in the field of fungal microorganisms and their role in sustainable agricultural practice could be gathered into three groups: AM fungi; P-solubilizing fungi, and fungi involved in bioremediation activities. The benefits of mycorrhizal fungi have gained significant attention in agricultural practices. The use of mycorrhizal and other fungal inoculants can improve crop yields, reduce the need for chemical fertilizers and pesticides, and enhance soil health but also facilitate plant resistance mechanisms against various stress conditions like salinity, drought, and temperature fluctuations and various types of toxicity. Thus, fungi also act as an essential part of the plant microbiome offering sustainable agriculture by ensuring ecosystem modulation and phytobiome engineering for successful crop production [22]. Fungi play a vital role in improving plant nutrition, particularly phosphorus acquisition. Phosphorus is a crucial macronutrient required for various cellular processes and energy production. However, it is often present in the soil in forms that are poorly accessible to plants. Fungi like *A. niger* boost the growth of plants, particularly offering a promising bio-input for vegetable seedling production. The emphasis of the scientific activity in the field of microbial fungal inoculants is on developing environmentally friendly and efficient microbial formulations and analyze how the introduced fungi affect microbial community, diversity, and the specific plant–microbial interactions, which determine the plant holobiome functioning [23].

## Data Availability

No new data were created.
